# Cytotoxic Effects of Some Flavonoids and Imatinib on the K562 Chronic Myeloid Leukemia Cell Line: Data Analysis Using the Combination Index Method

**DOI:** 10.4274/balkanmedj.galenos.2018.2017.1244

**Published:** 2019-02-28

**Authors:** Ferdane Danışman Kalındemirtaş, Hüsniye Birman, Eda Candöken, Sema Bilgiç Gazioğlu, Gülay Melikoğlu, Serap Kuruca

**Affiliations:** 1 Department of Physiology, İstanbul University İstanbul School of Medicine, İstanbul, Turkey; 2 Department of Biochemistry, İstanbul University İstanbul School of Pharmacy, İstanbul, Turkey; 3 Department of Immunology, İstanbul University Institute of Experimental Medicine, İstanbul, Turkey; 4 Department of Pharmacognosy, İstanbul University İstanbul School of Pharmacy, İstanbul, Turkey

**Keywords:** Cell culture, cell cytotoxicity, flavonoids, imatinib mesylate, K562 cells

## Abstract

**Background:**

Flavonoids are natural compounds with antioxidant, anticarcinogenic, and anti-inflammatory effects.

**Aims:**

To determine the cytotoxic effects of flavonoids and drug resistance related to P-gp on K562 human chronic myeloid leukemia cells. We also aimed to evaluate the therapeutic potential of imatinib and flavonoid combinations.

**Study Design:**

Cell culture study.

**Methods:**

In this study, K562 cells were treated with apigenin, luteolin, 5-desmethyl sinensetin and the anticancer drug imatinib mesylate. The effect of flavonoids on K562 cell proliferation was detected using the 3-(4,5-dimethylthiazolyl)2,5‑diphenyl‑tetrazolium bromide assay. Concentrations of apigenin, luteolin, and 5-desmethyl sinensetin ranging from 25 to 200 μM and of imatinib from 5 to 50 μM administered for 72 h were studied. Apoptosis/necrosis and P-gp activity were measured using flow cytometry. The combined effects of different concentrations of flavonoids with imatinib were evaluated according to combination index values calculated using CompuSyn software.

**Results:**

In our study, the IC_50_ values for apigenin, luteolin, and 5-desmethyl sinensetin were found to be 140 μM, 100 μM, and >200 μM, respectively. Luteolin (100 μM) had the highest cytotoxic activity of these flavonoids. These results were statistically significant (p<0.05). Among the flavonoids studied, the combination of luteolin and imatinib was the most effective and is therefore recommended for its cytotoxic activity in the K562 cell line. After 72 h of incubation at their respective IC_50_ concentrations, all flavonoids were associated with an apoptosis rate of approximately 50%. P-glycoprotein activity was increased in all groups. Combination treatment may provide better outcomes in terms of cytotoxicity and thus reduce the dosages of imatinib used.

**Conclusion:**

The combination of some flavonoids and imatinib mesylate may increase the cytotoxic effect; However, the antagonistic effect should be considered in combined use on k562 cells.

Flavonoids are a group of natural plant metabolites found in a variety of fruits and vegetables. Although the effects of flavonoids have been investigated for many years, their antioxidant, anticarcinogenic, and anti-inflammatory effects have only recently gained recognition (1,2,3). Flavonoid compounds also have a wide spectrum of pharmacologic activities and can serve as chemopreventive agents (4,5).

*In vivo*,* in vitro*, and epidemiologic studies revealed that flavonoids have various antioxidant, anti-thrombotic, antihypertensive, antiallergic, anti-inflammatory, and anti-apoptotic bioactivities (6,7).Studies have been conducted recently on the anticancer activities of various natural compounds, in particular those of flavonoids. The protective and therapeutic effects of flavonoids such as kaempferol, quercetin, luteolin, and apigenin have been reported (8).

A strong correlation was reported between flavonoid intake and the prevention of chronic diseases such as cancer. Flavonoids have anticancer effects on breast and prostate cancers because the cells of these cancers synthesize high levels of hormone receptors (9).

Chronic myeloid leukemia (CML) is a chronic, myeloproliferative, and malignant disease of bone marrow stem cells. Myeloid cells are capable of differentiation, but in CML there is uncontrolled proliferation (10).Although treatment with tyrosine kinase inhibitors has led to significant improvements in CML outcomes, drug resistance may be seen as a major problem with their use. Various studies have been conducted to overcome drug resistance (11,12,13,14).

P-glycoprotein (P-gp) is as a carrier protein encoded on the seventh chromosome (7q21) by the MDR 1 gene. P-gp is a member of the ATP-dependent membrane transport proteins, which reduce intracellular drug concentrations by pumping foreign substances from cells (12,15). In excess, P-gp expression can develop resistance to chemotherapeutic drugs. Many human tissues have P-gp. P-gp protects the hematopoietic system against endogenous toxic compounds and xenobiotics (15,16,17).

In this study, our purpose was to examine the cytotoxic and apoptotic effects and P-gp drug resistance on K562 CML cells. We also aimed to evaluate the therapeutic potential of drug and flavonoid combinations.

## MATERIALS AND METHODS

### Drugs

Apigenin and luteolin were purchased from Sigma (Sigma 42251, 72511, St. Louis, USA), and 5-desmethyl sinensetin was purified by thin layer chromatography and paper chromatography from *Artemisia austriaca *Jacq. in İstanbul University Faculty of Pharmacy, Department of Pharmacognosy. Dimethyl sulfoxide (DMSO) (DMSO; Sigma-Aldrich D2650) was used as a diluent for stock solutions of apigenin, luteolin, and sinensetin, which were aliquoted and stored in the dark at -20 °C until use, then diluted with medium.

### Cell culture

The leukemia cell line K562 was purchased from the American Type Culture Collection (ATCC, Manassas, VA). Cells were maintained in Roswell Park Memorial Institute Medium (RPMI 1640; Gibco) supplemented with 10% heated-inactivated fetal bovine serum (FBS; Sigma), 100 units/mL penicillin, and 100 μg/mL of streptomycin (Gibco 15140-122) in a humidified incubator with an atmosphere of 5% CO_2_ at 37 °C. In order to produce a sufficient cell number for tests, cells were passaged after they reached 80% monolayer confluency. K562 cells grown in RPMI 1640 were exposed to increasing concentrations of imatinib (1-25 μM), luteolin (25-200 μM), apigenin (25-200 μM), and 5-desmethyl sinensetin (25-200 μM). The cells were also exposed to combinations of flavonoids and imatinib at IC_50_ and lower doses (Table 1 and Table 2).

### MTT assay

In our study, any cytotoxic effect was determined by using a 3-(4,5-dimethylthiazolyl)2,5‑diphenyl‑tetrazolium bromide (MTT) assay. The cells were cultured in 96-well plates with 3×10^5^ cells/100 μL in each well. Briefly, 10 μL/well of 50, 100, 150, and 200 μM concentrations of apigenin, luteolin, 5-desmethyl sinensetin, and (as positive control) imatinib (5, 10, 25, 50 μM) were added, and cells (90 μL/well; 10^5^ cells/mL culture medium) were subsequently seeded and incubated for 72 h. In the same way, K562 cells were also exposed to combinations of flavonoids and imatinib at the doses outlined in Table 2. After subsequent incubation with MTT (Sigma-Aldrich) solution (10 μL of 5 mg/mL PBS) at 37 °C for 4 h, half of the medium was removed from each well, and the cells were lysed with dimethyl sulfoxide, in which formazan crystals formed by MTT dissolved. The absorbance was read in an enzyme-linked immunosorbent assay plate reader (Rayto, China) at 570 nm, with a reference wavelength of 620 nm. All experiments were repeated at least three times.

### Apoptosis/necrosis determination

The amount and location of phosphatidylserine in normal, apoptotic, and necrotic CML cells were evaluated using an annexin V-FITC/propidium iodide assay kit (Millipore, USA) according to the manufacturer’s instructions. Chronic myeloid leukemia cells were cultured at 1×10^6^ cells in 2 mL medium in every well of a 6-well plate. Apoptosis and necrosis evaluation was performed at the respective IC_50_ doses and for 72 h to match the MTT assay as follows: 140 μM apigenin, 100 μM luteolin, and 5 μM imatinib, and >200 μM sinensetin. For combination treatments, the following concentrations were used: imatinib (2.5 μM) + luteolin (50 μM), imatinib (2.5 μM) + apigenin (70 μM), and imatinib (2.5 μM) + sinensetin (100 μM). For this reason, IC_50_ concentrations of apigenin, luteolin, and 5-desmethyl sinensetin and their combinations with imatinib were added to each well and incubated in a 5% CO_2_ atmosphere for 72 hours at 37 °C. Cells were subsequently transferred to falcon tubes 15 mL and centrifuged for 10 minutes. The cell pellet was homogenized with 1 mL PBS and centrifuged again after a 100 μL sample was removed from this mixture. The newly formed cell pellet was homogenized in annexin binding buffer and stained with 5 μL annexin V-FITC/propidium iodide. The cell suspension was incubated at room temperature in the dark for 15 minutes. Data were acquired with a FACS Calibur flow cytometer and analyzed with CELL QUEST software (BD Biosciences). To assess apoptosis and necrosis, the IC_50_ was applied for 72 h to match the conditions of the MTT assay.

### P-glycoprotein determination

Chronic myeloid leukemia cells were cultured at a density of 1×10^6^ cells in 2 ml medium in every well of a 6-well plate. Quantitation of P-gp was performed at the following IC_50 _doses and for 72 h to match the conditions of the MTT assay: 140 μM apigenin, 100 μM luteolin, 5 μM imatinib, and >200 μM sinensetin. For combination assays, the following concentrations were used: imatinib (2.5) + luteolin (50), imatinib (2.5) + apigenin (70), and imatinib (2.5) + sinensetin (100). For this reason, IC_50_ concentrations of apigenin, luteolin, and 5-desmethyl sinensetin were added to each well and incubated in a 5% CO_2_ atmosphere for 72 h at 37 °C. At the end of the incubation period, the cells from each well were transferred to separate Falcon tubes. Each tube was centrifuged for 5 minutes at 1500 rpm, and the supernatant was removed. Two milliliters of cell washing solution was added to the remaining pellet, and the tubes were centrifuged for 5 minutes at 1500 rpm for a second time. The supernatant was once again removed, and 500 μL cell washing solution was added. Subsequently, 2 μL MDR 1 PE (Santa Cruz, USA) was added to this mixture, which was then incubated at room temperature in the dark for 45 minutes. After these steps, cell suspensions were analyzed using a FACS Calibur model (BD Bioscience) flow cytometer.

### Statistical analysis

Data were tested for normality using the Shapiro-Wilk test and found to be normally distributed; therefore, parametric tests were applied. The groups were compared using one-way analysis of variance. Tukey’s post-hoc test was used for multiple comparisons due to homogeneity of variances. Differences were considered significant when p<0.05. Analyses were performed using GraphPad software. The combined effects of different concentrations of flavonoids with imatinib were evaluated, and the combination index (CI) values were determined using CompuSyn software.

## RESULTS

### Effects of apigenin, luteolin, sinensetin flavonoids, and imatinib on K562 cells

The dose- and time-related antiproliferative effects of apigenin, luteolin, 5-desmethyl sinensetin, and imatinib mesylate were assessed on K562 human chronic myeloid leukemia cells.

These results were evaluated using an MTT assay at the end of 72 h (Table 1).

### Combined effects of apigenin, luteolin, and 5-desmethyl sinensetin flavonoids with imatinib on K562 cells

The effects of different concentrations of flavonoids in combination with imatinib were evaluated for cytotoxicity. The combined cytotoxic effect was found to be greater than that of either flavonoids or imatinib when used alone (Table 2). Four different concentrations of each flavonoid in combination with imatinib were evaluated. The results of comparisons between 12 different groups are shown in Table 2. The CI was calculated using CI equation algorithms (18,19,20) with CompuSyn software. CI= 1, <1, and >1 indicates an additive effect, synergism, and antagonism, respectively.

A slightly synergistic effect was detected for CI values calculated using the CompuSyn Report program when luteolin and imatinib IC_50_ were combined in half doses, and there was a synergistic effect under the same conditions when the luteolin dose was decreased. Antagonism and near antagonism were detected when the imatinib dose was decreased (Table 2).

However, there was a slightly synergistic effect of the combination of apigenin and imatinib at half of the IC_50_ values (Api: 70, Im: 2.5 μM), and a strong antagonistic effect was observed when apigenin was decreased to 35 μM. Near antagonism and antagonism were detected when the dose of imatinib was decreased to 1.25 μM.

Finally, antagonism was detected when half (Sin: 100, Im: 2.5 μM) and one quarter (Sin: 50, Im: 1.25 μM) of the IC_50_ values were used in the combination of sinensetin and imatinib. The dose-reduction index (DRI) was calculated from the DRI equation and an algorithm using CompuSyn software. DRI=1, >1, and <1 indicate no dose reduction, favorable dose reduction, and unfavorable dose reduction, respectively, for each drug in the combination. These results are summarized in Table 2.

**Figure 1:**
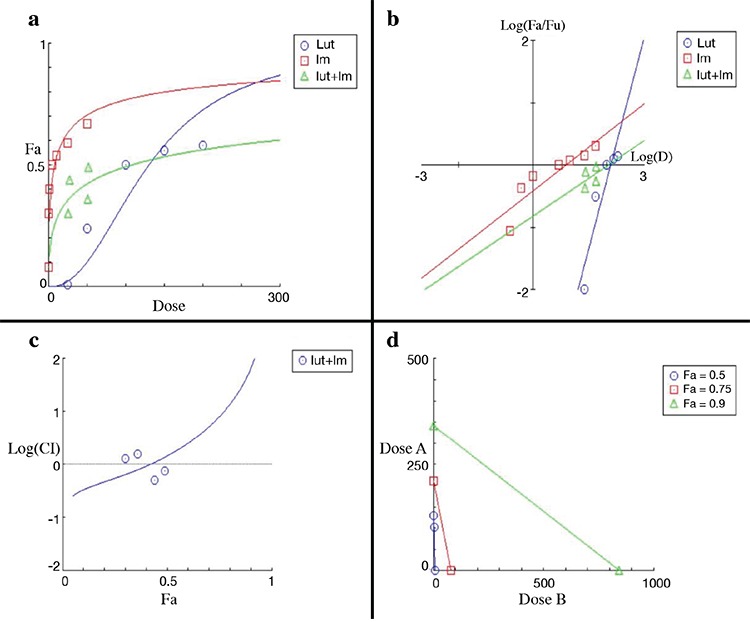
Graphic representations obtained from the CompuSyn report for luteolin and imatinib combinations. Dose-effect curve (a). Median-effect plot (b). Logarithmic combination index plot (c). Isobolograms (d). Fa: fraction affected

The combined effect of luteolin and imatinib is shown in Figure 1. When we used imatinib and luteolin together, we obtained cytotoxicity with lower luteolin and imatinib concentrations than when we used them individually (Figure 1). The isobolograms obtained at a fraction affected of 0.5, 0.75, and 0.9 are shown in Figure 1d.

The CI was a function of the fraction affected. The fraction affected by concentration increased from 0 to 1 (18,19,20,21).

**Figure 2:**
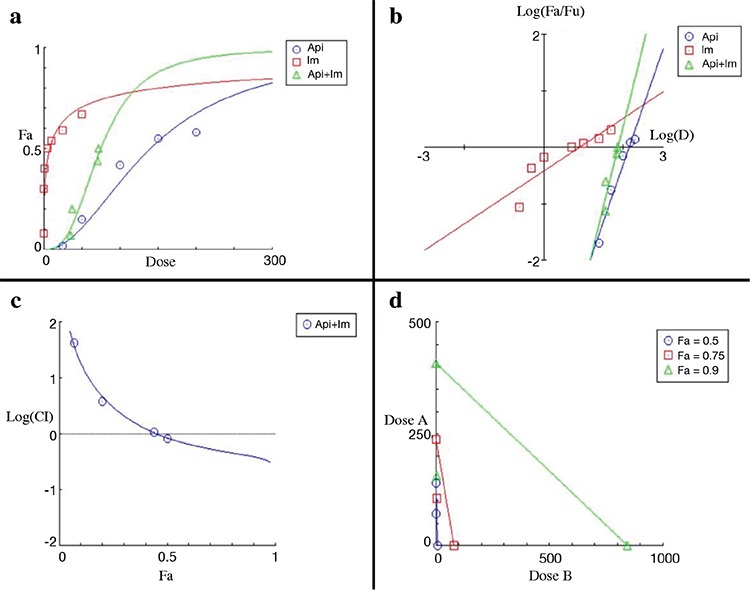
Graphic representations obtained from the CompuSyn report for apigenin and imatinib combinations. Dose-effect curve (a). Median-effect plot (b). Logarithmic combination index plot (c). Isobolograms (d). Fa: fraction affected

The synergistic effect of apigenin and imatinib according to CompuSyn quantification is shown in Figure 2.

**Figure 3:**
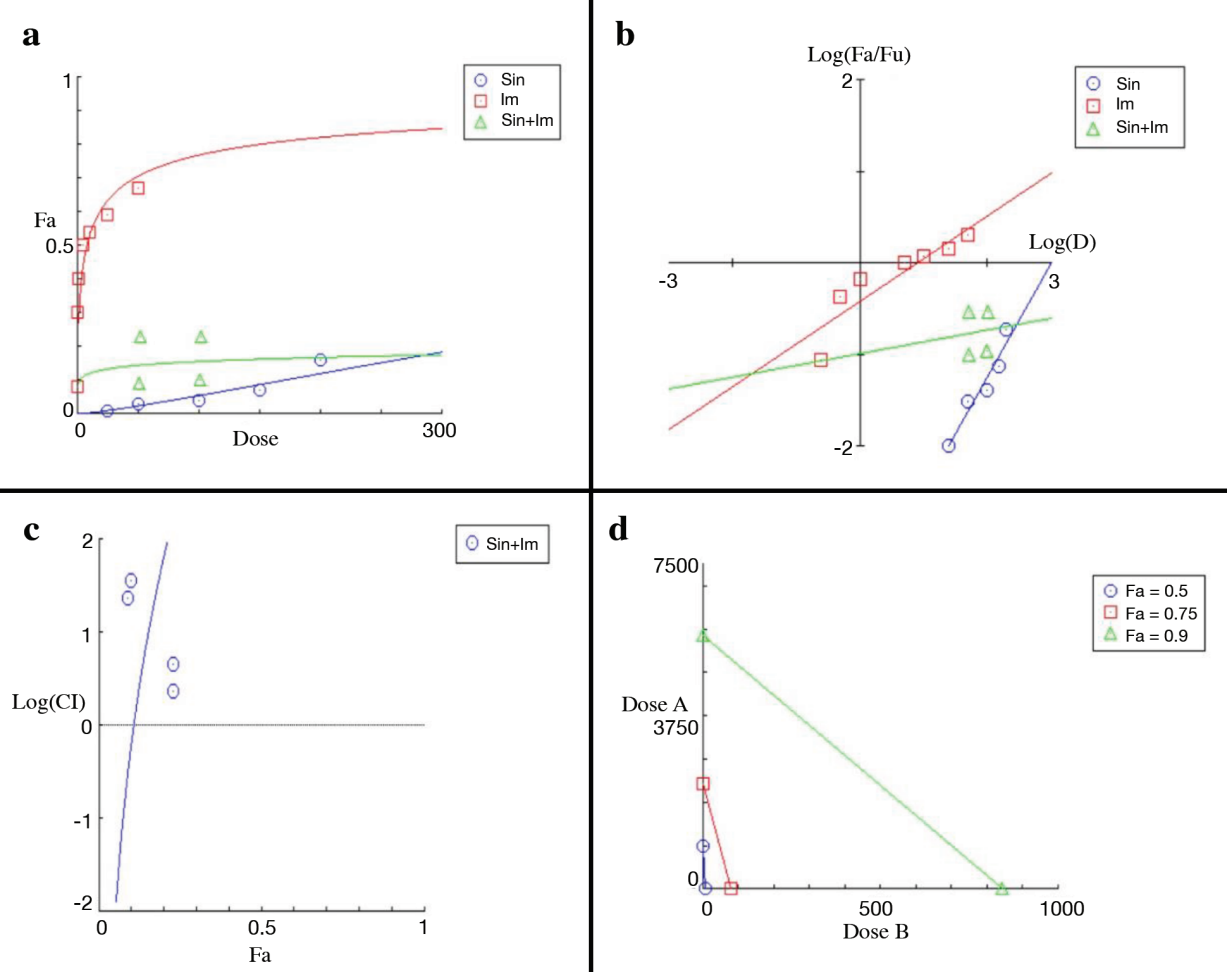
Graphic representations obtained from the CompuSyn report for sinensetin and imatinib combinations. Dose-effect curve (a). Median-effect plot (b). Logarithmic combination index plot (c). Isobolograms (d). Fa: fraction affected

With reference to Figures 1, 2, and 3 and Table 2, the combined use of luteolin and imatinib (Figure 1) showed more synergistic results than apigenin and imatinib (Figure 2). Antagonistic effects were observed with the combination of imatinib and 5-desmethyl sinensetin (Figure 3).

### Apoptosis-necrosis and the expression of P-gp in K562 cells

**Figure 4:**
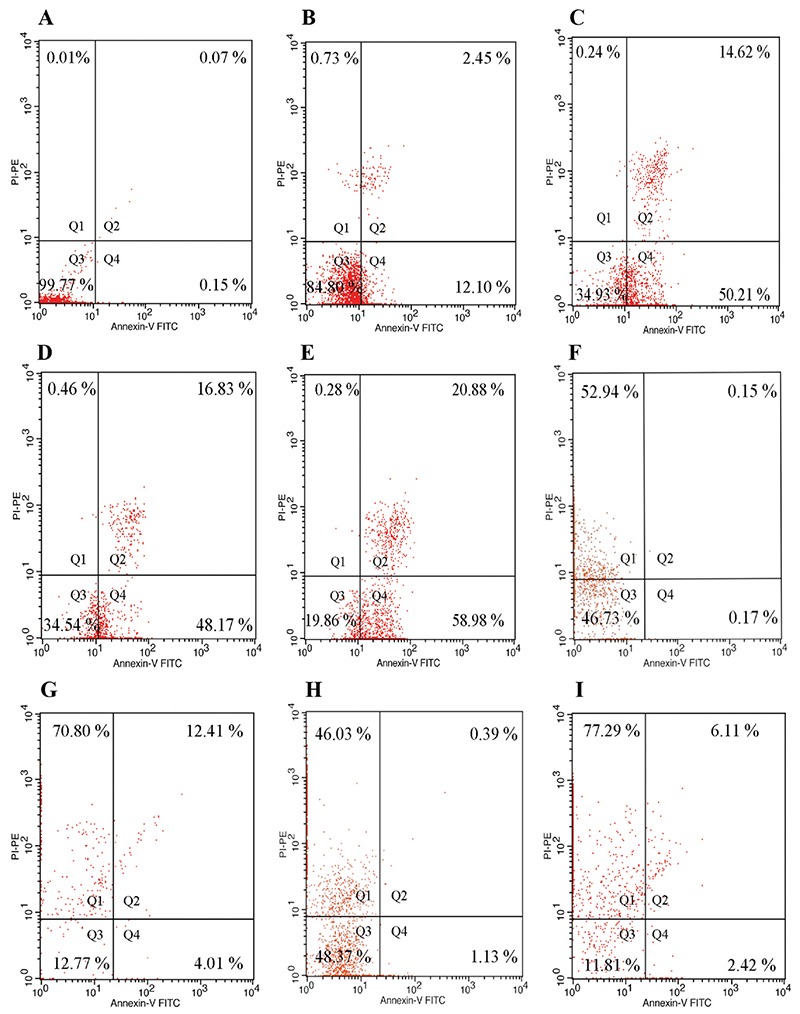
Increase in apoptosis and necrosis with flavonoid treatment. K562 cells incubated for 72 hours with flavonoids and imatinib, stained with annexin V and propidium iodide. The proportion of cells stained alone and with annexin V, propidium iodide alone, and annexin V + propidium iodide. Without stained control (a). Control (b). Apigenin (c). Luteolin (d). 5-desmethyl sinensetin (e). Imatinib (f). Apigenin + imatinib (g). Luteolin + imatinib (h). Sinensetin + imatinib (i). PI: propidium iodide

**Figure 5:**
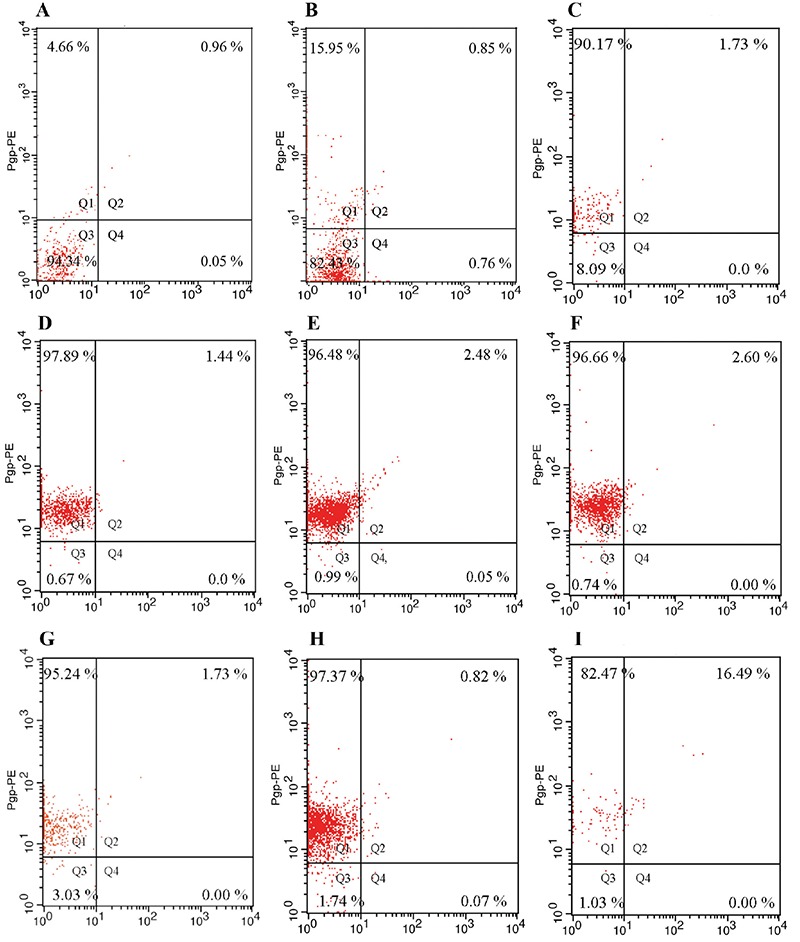
Distribution of P-glikoprotein + K562 cells stained with phycoerythrin conjugated P-glycoprotein after 72 hours incubation with imatinib, flavonoids and combinations of imatinib with flavonoids. Without stained control (a). Control (b). Apigenin (c). Luteolin (d). 5-desmethyl sinensetin (e). Imatinib (f). Apigenin + imatinib (g). Luteolin + imatinib (h). Sinensetin + imatinib (i). Pgp-PE: phycoerythrin conjugated P-glycoprotein

The effects on apoptosis and P-gp activity after incubation of K562 cells with apigenin, luteolin, sinensetin, and imatinib in various combinations at IC_50_ concentrations for 72 hours are depicted in Figure 4 and 5, respectively.

## DISCUSSION

In this article, the dose-dependent cytotoxic effects of apigenin, luteolin and 5-desmethyl sinensetin (5-hydrochyl-6,7,3’,4’-tetramethoxyflavone) on K562 cells were investigated. In addition, imatinib mesylate, an anticarcinogenic drug, was used as a positive control and investigated in combination with flavonoids. Cytotoxic activity is expressed as IC_50_ values, which are the concentrations that inhibit 50% of cell growth.

In our study, the IC_50_ values for apigenin, luteolin, and 5-desmethyl sinensetin were found to be 140 μM, 100 μM, and >200 μM, respectively. The IC_50_ value for imatinib was 5 μM. Luteolin (100 μM) was found to be the most effective when we compared the cytotoxic activities of these flavonoids. However, these values were higher than those of imatinib. Hence, combinations were designed as the 2^nd^ phase of the study.

In recent years, many different combined compounds and therapeutic aproaches have been used to treat devastating diseases such as cancer and AIDS. The main aim is to achieve a synergistic therapeutic effect, to reduce the dose and toxicity, and to minimize or delay the induction of drug resistance such that the combinations would have added benefits (19,20). In *in vitro* studies, the CI method is a quantitative representation of the pharmacologic interaction between two drugs. Several important and clinically relevant interactions between flavonoids and conventional drugs have been reported over the last few years (21).

Luteolin and imatinib combinations were the most effective and recommended for their cytotoxic activity in the K562 cell line among the flavonoids that we studied. Thus, when flavonoids are administered together with imatinib, we believe that effective treatment may be provided at a lower dose of imatinib.

Alternative therapeutic methods are investigated for their potential utility in cancer prevention and treatment. The combined use of herbal flavonoids with cytotoxic effects and various anticancer drugs has previously been targeted, an approach that could lead to reduced dosing of anticancer drugs (19,20,21). One of the approaches to increasing the therapeutic efficacy of therapy is to add natural compounds to sensitize cells to the cytotoxic activity of drugs. However, there are not enough published preclinical studies on this method. Therefore, many drugs are in use for the treatment of various cancers, and natural phytochemicals such as flavonoids are widely used in cancer chemoprevention. Combination treatment may provide better outcomes and reduce the dosage of imatinib required for cytotoxicity.

The aim of more effective treatment is to use lower doses of drugs and shorter incubation periods. Extensive studies have been performed in this area (21,22,23). The findings of these studies suggest that combinations may be effective for the treatment of leukemia. The IC_50_ value of apigenin was found to be higher than that of luteolin, and a further antagonistic effect was detected according to the CI (CI >1). We concluded that apigenin had lower cytotoxicity than luteolin; flavonoid 5-desmethyl sinensetin had no effective cytotoxic activity against IC_50_ and CI values.

Although a synergistic effect (Im: 2.5 μM) was observed in the combination of luteolin and imatinib, antagonistic and near-antagonistic effects were observed when the imatinib dose was reduced (Im: 1.25 μM). A strong antagonistic effect was observed in the combination of apigenin and imatinib (Api: 35 μM). Thus, our study shows that the antagonistic effect of flavonoid compounds and imatinib, an anticancer drug, is an important finding to be considered. This result may affect CML treatment with the use of flavonoid-containing compounds.

Epidemiologic studies reported a high correlation between flavonoid intake and prevention of chronic diseases such as cancer (24,25). Several mechanisms for the antiproliferative activity of flavonoids on cancer cells have been proposed. In particular, these compounds may be effective in the induction of cell cycle arrest and apoptosis (24,26). Leopoldini et al. (27) reported that the number and positions of hydroxy groups were important for the antioxidant activity in flavonoids. We also demonstrated that the cytotoxic activity of sinensetin, which has methoxyl groups, was lower than that of other flavonoids. It has previously been shown that methoxyl groups might have higher cytotoxic activity, but our data did not support this finding for the flavonoid 5-desmethyl sinensetin.

After incubation at their respective IC_50_ concentrations, all flavonoids had an approximately 50% apoptotic effect on the cells. These findings indicated that cytotoxic activity might have occurred through an apoptotic mechanism, in contrast with imatinib and combinations thereof. When we used imatinib alone and in combination with our flavonoids, necrosis appeared at increasing rates after these cells were incubated for 72 hours. The IC_50 _value of imatinib is relatively low compared with than that of flavonoids, which cause cell death by necrosis. There is also evidence to support a necrotic effect of imatinib (28,29); however, other studies found that imatinib causes cell death by apoptosis (30,31). Imatinib in combination with flavonoids increased the necrosis rate despite the 50% decrease in the concentration applied. Researchers have different ideas about which is the preferable mechanism of cancer cell death in effective chemotherapy (32); apoptosis is programmed cell death in which no damage is caused to healthy tissue, whereas necrosis damages surrounding tissue, which causes inflammation. Other authors stated that necrosis contributes to the fight against cancer by stimulating the immune system of the organism by providing macrophage and lymphocytes migrate to the inflammatory areas (33).

According to our results, re-assessment of the clinical use imanitib is required, because if necrosis is preferred to stimulate the immune system, or apoptosis is preferred to avoid systemic damage and an inflammatory response, this may be an important clinical choice. Apigenin, luteolin, and 5-desmethyl sinensetin also triggered the apoptosis pathway. Therefore, this findings are open to discussion.

In recent years, development of drug resistance has become one of the most challenging problems for many patients, despite the therapeutic effects of drugs (34). It is known that cancer cells that are resistant to any agent may show cross-resistance to others. However, the important point in the present study is that K562/IMA-3 (resistant cells) were approximately 4.25-fold more resistant to apigenin as compared with sensitive cells at 48 h; IC_50_ values of apigenin were higher in resistant cells than in sensitive cells (11).

As for our other findings, in contrast to many previous studies, it was observed that P-gp activity increased after 72 h of incubation of K562 cells with flavonoids and imatinib at their respective IC_50 _values. We detected initial P-gp activity values in K562 cells of 15.72%, and the IC_50_ values were high. The high P-gp and IC_50_ values show that K562 cell lines developed spontaneous resistance. P-gp activity was not at a low level at the beginning of our study. After incubation with flavonoids and imatinib, the cells acquired resistance (Figure 4).

We used the IC_50 _value for flavonoids and imatinib to detect P-gp expression. Although the concentration of a substance that inhibits the growth of 50% of the cells (IC_50_) must be significantly effective, this concentration also has a toxic effect. Chronic myeloid leukemia cells may also acquire resistance to imatinib by different mechanisms, such as P-gp overexpression (35,36). Some researchers have obtained different results with different flavonoids (37,38).

As expected, we found that P-gp increased in all groups. This transport protein provides a route of efflux from the cell to maintain cell viability. Thus, as P-gp levels increased, the rate of drug efflux would also have increased. We believe that this effect is related to increased expression and activity of proteins that transport substances out of cells, resulting in increased P-gp activity. P-gp is likely a carrier protein in the transport of substances out of the cells. Therefore, increased P-gp activity appears to be a probable mechanism aimed at cellular preservation.

The cause of increased P-gp (50 μM and 72 h) may have been the high dose and long incubation with flavonoids. We suggest that incubation of cells over shorter periods will be useful for understanding the mechanism by treatment of cells with drugs. Previous studies support our results: Souza et al. (39) observed that high-dose vincristine induced concomitant overexpression of P-gp and survivin.

Combination imatinib with flavonoids can provide protection against drugs toxicity and side effects and support the immune system by different mechanisms (40).

We believe that physicians should avoid extended use of flavonoids for anticancer purposes, and drug resistance must be carefully evaluated, at least in K562 cells. More studies must be conducted on this topic in the future.

As a conclusion, this study indicates that the combination of flavonoids and imatinib mesylate were able to enhance the cytotoxic effect on K562 cells. An advantage of using drug combinations is that lower concentrations of anticancer drugs can be used because of each drug can act through different mechanisms. Otherwise, P-gp levels may be increased by exposing cells to high concentrations of drugs. These results suggest that flavonoids and imatinib mesylate in combination could enhance the cytotoxic effect, but in the combination of imatinib and flavonoids, the antagonistic effect of some doses should be considered. More studies must be conducted on this topic in the future.

## Figures and Tables

**Table 1:**
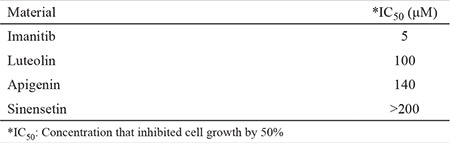
Determination of IC_50_ values of favonoids on a human chronic myeloid leukemia cell line (K562) incubated with various concentrations of flavonoids: apigenin, luteolin, sinensetin, and imatinib

**Table 2:**
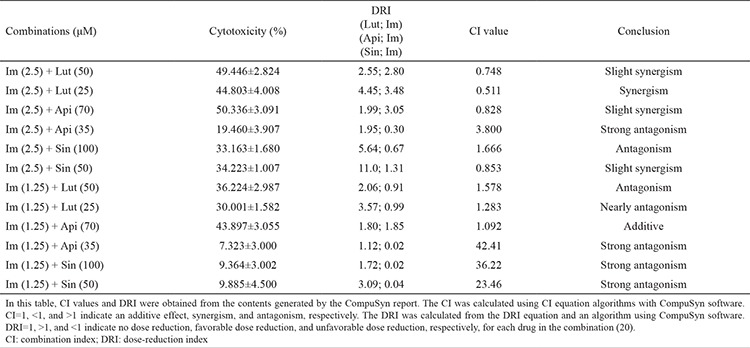
The combined effects of apigenin, luteolin, and 5-desmethyl sinensetin with imatinib on human chronic myeloid leukemia cells after 72 hours of incubation
